# Characterisation of *Pasteurella multocida* Strains from Different Lesions in Rabbits

**DOI:** 10.3390/ani14111569

**Published:** 2024-05-25

**Authors:** Francesco D’Amico, Davide Messina, Gaia Casalino, Michele Schiavitto, Antonella Bove, Diana Romito, Francesco Paolo D’Onghia, Antonio Camarda, Elena Circella

**Affiliations:** 1 Department of Veterinary Medicine, University of Bari, S. P. Casamassima km 3, 70010 Valenzano, BA, Italy; francesco.damico@uniba.it (F.D.); antonella.bove@uniba.it (A.B.); diana.romito@uniba.it (D.R.); francescopaolo.donghia@uniba.it (F.P.D.); antonio.camarda@uniba.it (A.C.); elena.circella@uniba.it (E.C.); 2 Division of Veterinary Clinical Science, School of Veterinary Medicine and Science, Faculty of Medicine and Health Sciences, University of Nottingham, Sutton Bonington Campus, Loughborough LE12 5RD, UK; davide.messina1@nottingham.ac.uk; 3 Italian Rabbit Breeders Association, ANCI, Contrada Giancola snc, 71030 Volturara Appula, FG, Italy; micheleschiavitto@anci-aia.it

**Keywords:** *Pasteurella multocida*, serogroup, virulence genes, pasteurellosis, *Oryctolagus cuniculus*

## Abstract

**Simple Summary:**

The aim of this study is to provide information about the *Pasteurella multocida* strains that are responsible for pasteurellosis in rabbits. A total of 114 strains responsible for different lesions in rabbits from industrial farms were investigated. In detail, the serogroup belonging to and the presence of 15 virulence-associated genes were investigated using PCR (polymerase chain reaction) protocols. The tested strains belonged to serogroups A, D, and F. Type A was the most frequently identified and it was prevalently found in strains responsible for rhinitis and pneumonia. Type D was the prevalent capsular type of strains responsible for metritis, while F was identified in strains detected from otitis, mastitis, subcutaneous abscesses, and septicaemia. Among the virulence-associated genes, *sodC* was found in all tested strains. Gene *pfhA* was more frequently found in strains belonging to type A than in type D and it was prevalently found in strains responsible for respiratory lesions. In addition, it was found in all F strains, suggesting a strong relationship between *pfhA* and this capsular type. Conversely, gene *tadD* was rarely found in strains belonging to capsular type F. Gene *fur* occurred more frequently in strains belonging to type D and involved with rhinitis. Gene *hgbB* was detected prevalently in strains responsible for metritis. Additionally, based on the results, the presence of more than 8 virulence-associated genes in the strains seems to increase the risk of the occurrence of severe lesions in the affected rabbits. Nevertheless, the detection of some strains responsible only for rhinitis which were equipped with 10 and 11 virulence-associated genes, seems to emphasise the importance of a gene’s expression rather than its presence in strains.

**Abstract:**

Pasteurellosis, a disease caused by *Pasteurella multocida*, is responsible for economic losses in rabbit industrial farms due to rhinitis, conjunctivitis, pneumonia, metritis, mastitis, orchitis, subcutaneous abscesses, otitis, encephalitis, and septicaemic forms. Although the occurrence of the disease is conditioned by predisposing factors that affect the rabbit immune response, the strains of *P. multocida* involved in the infection may have a different pathogenic ability. Therefore, typing of strains spread among the rabbits is important to assess their pathogenic potential. The aim of this study is to investigate the *P. multocida* strains responsible for disease in rabbit industrial farms. A total of 114 strains identified from different lesions were serotyped. Additionally, the presence of virulence-associated genes was investigated using three PCR (polymerase chain reaction) protocols. Capsular type A was prevalently found in strains from respiratory lesions while types D and F in those from metritis, mastitis, and other lesions. Different associations between some virulence-associated genes and both capsular type and lesions found in rabbits were detected. The presence of 8 virulence-associated genes seems to increase the occurrence of metritis. In addition, strains belonging to capsular type A and responsible for respiratory disorders especially, were found equipped with 10 and 11 virulence-associated genes. Nevertheless, the presence of strains responsible only for rhinitis was also detected among the latter, suggesting that the pathogenic ability of the bacteria depends on the expression rather than the presence of a gene.

## 1. Introduction

*Pasteurella multocida* (*P. multocida*) is a potential zoonotic pathogen, which can cause respiratory disorders in different hosts [1]. *Pasteurella multocida* is classified into five capsular types (A, B, D, E, and F), which are further classified into 16 Heddleston serovars (1–16), based on lipopolysaccharide (LPS) antigens [2]. Capsular types A, D, and F have been reported for rabbit isolates [3]. Usually, strains possessing the capsule are more virulent than acapsular variants [4]. The associations between serovars and disease in specific hosts have been reported. Serovars A and F are involved in respiratory diseases and fowl cholera; serovars B and E in haemorrhagic septicaemia in ungulates and serovar D is usually isolated in cases of atrophic rhinitis of swine [5,6,7]. Pasteurellosis of rabbit is prevalently associated with capsular type A and, less frequently, with types D and F [3,8]. In addition, types B and F can be highly pathogenic in rabbits if associated with capsular types A and D particularly [9,10].

The variability of *P. multocida* strains involved in the infection is also expressed on the clinical pictures associated with the disease that may include different pathological conditions in rabbits, represented by respiratory tract pathologies prevalently, but also otitis, pyometra, mastitis, orchitis, abortions, subcutaneous abscesses, and acute septicaemic forms [11]. *P. multocida* infection in rabbits is endemic, with an estimated prevalence of infection ranging from 7% to 100% [12]. Although the microorganism is highly infectious, rabbits may have often subclinical infection for long periods or may be asymptomatic being carriers of the germ. The association with *B. bronchiseptica* in the respiratory tract of both healthy and sick animals can occur [13]. The disease in farms particularly occurs when stress, concomitant pathologies or unfavourable environmental conditions occur. The most important managerial factors that can influence the virulence of *P. multocida* are dustiness, non-optimal temperatures and humidity, airspeed, and ammonia, which should not exceed the limit of 25 ppm in rabbit farms [14]. The transmission of the pathogen occurs through direct contact between animals suffering from acute forms by the aerosol, or indirectly through fomites [15]. Another important vehicle for the transmission of *P. multocida* in intensive farms is represented by infected semen and contaminated material used for artificial insemination, which can carry the germ into the female genital tract. Considering the anatomical conformation of the reproductive apparatus of the rabbit, the insemination pipette can deeply introduce the germ and, if handled incorrectly, damage the mucosa [16]. Epidemiologically, the healthy carriers play a decisive role in maintaining the infection within the flock, among the does particularly, which may develop chronic infections that may affect their reproductive capacity with a significant economic impact [16]. In addition, at the end of gestation, physiological modifications cause an immunosuppression lasting a few days which can favour the exacerbation of the pre-existing pathological condition with the onset of respiratory disorders, which represent the most important cause of death in breeding stock [17]. The biosecurity measures adopted in farms have an important role in reducing the spread of the pathogen. In addition, the immunisation prophylaxis is also used in many countries, including Italy. However, the protective efficacy of mainly commercially available vaccines is not completely satisfactory, especially against heterologous strains [18]. In Italy, although a commercial vaccine against *P. multocida* is available for use in rabbit farms, the Ministry of Health authorises the use of stabulogenic vaccines prepared using *P. multocida* strains isolated from sick/dead rabbits for farms in which pasteurellosis recurrently occurs. Considering that different serotypes of *P. multocida* can be found in the same farm, even in different periods, the monitoring of the serotypes spread in rabbit flocks prior to the formulation of each new vaccine batch of stabulogenic vaccine is important to match the major possible potential efficacy. *P. multocida* strains may differ also based on their pathogenicity which is associated with various virulence factors (VFs) in rabbits [7]. The most important ones have been identified in capsule proteins and lipopolysaccharides [19]. In addition, many other virulence genes encoding for fimbriae, adhesion, and colonisation factors (*ptfa*, *fimA*, *pfhA* e *tadD*), iron regulation factors and protein acquisition (*exbB*, *exbD*, *tonB*, *hgbA*, *hgbB*, *tbpA*, *fur*), superoxide dismutase (*sodA* and *sodC*), dermonecrotic toxins (*toxA*), a variety of outer membrane protein (OMPs) as protective factors (*ompA*, *ompH*, *omp87*, *plpB*), and neuraminidase (*nanB* and *nanH*) are considered to be important [7,9,20,21]. These virulence factors facilitate the colonisation and tissue invasion of *P. multocida* through the evasion of host defence mechanisms, tissue destruction, and stimulation of the inflammatory response [22]. The relationship between some VFs and capsular types has been highlighted. In fact, type A has greater adhesion capability on the respiratory mucosa than type D which instead has the production of dermonecrotic exotoxins as the main pathogenetic mechanism [7]. Genes encoding for virulence factors are considered as markers for the definition of the pathogenic potential of *P. multocida* and the genetic characterisation may be useful to discriminate the different strains involved in the infection [23]. In addition, some virulence factors have been considered as potential candidates for the preparation of vaccines [24].

The aim of this study was to investigate *P. multocida* strains isolated from rabbits affected by pasteurellosis by assessing the serogroup they belong to, the presence of genes associated with virulence, and the possible relationship between serogroup and pattern of virulence genes and the clinical form found in the rabbits.

## 2. Materials and Methods

### 2.1. Origin of the Bacterial Strains Isolated

One hundred and fourteen strains of *P. multocida* were investigated. The strains were isolated from fattening rabbits and does, housed in nine industrial farms and affected by pasteurellosis, over a seven-year period, from 2017 to 2023. *P. multocida* strains were either isolated from nose of affected rabbits, based on clinical signs and using sterile swabs moistened in sterile saline solution (0.9%), or from different tissues, lung, ear, subcutaneous abscess, udder, blood from heart, uterus, based on pasteurellosis-compatible lesions revealed by necropsy. 

All samples were plated on Tryptic Soy Agar (TSA) (OXOID, Basingstoke, UK) supplemented with 5% sheep blood and incubated at 37 °C for 24 h under aerobic conditions.

From each sample, three to four colonies morphologically compatible with *Pasteurella* spp. (greyish, translucent, non-haemolytic) were individually plated on TSA enriched with 5% blood for 24 h to obtain pure replication. The Multiplex PCR according to [25] with some modifications was used to obtain the identification as *P. multocida* and contextually to define the capsular type. Briefly, the colonies were diluted in 0.6 mL microtubes containing 100 µL of sterile distilled water and DNA extraction was performed by boiling them for 10 min.

The PCR was performed in a mixture consisting of 12.5 µL of 1X Platinum Mastermix (Thermo Scientific, Milan, Italy) and containing 0.2 µL and 0.3 µL (50 pmol/µL primary concentration) of primer pairs for species identification and capsular types, respectively, and ultra-pure nuclease-free water (Thermo Scientific) until a final volume of 25 µL was reached. Cycling conditions were: 95 °C for 5 min, 35 cycles, each with 95 °C for 30 s, 55 °C for 30 s, 72 °C for 1 min and 10 s, and a final extension at 72 °C for 10 min. The PCR products were loaded for electrophoresis using a 1.5% agarose gel, which was stained with ethidium bromide. The reaction was visualised using the Gel Doc-It image analyser (UVP, Upland, CA, USA). Each strain identified as *P. multocida* was stored at −80 °C in brain heart infusion with glycerol (20%).

### 2.2. Pathogenicity Genes Investigation

Each strain was plated on Tryptic Soy Agar (TSA) supplemented with 5% sheep blood and incubated at 37 °C for 24 h under aerobic conditions. Eleven strains among those stored for longer time, not having grown or having had difficulty in growing, were discarded. Therefore, 103 strains were investigated for virulence-associated genes.

The DNA extraction was performed as already described before.

Three Multiplex-PCR protocols, named A, B, and C, previously described [26] were used to investigate 15 different genes encoding for potential virulence factors of *P. multocida*. Briefly, the PCR mixture for each Multiplex-PCR consisted of 12.5 µL of 1× Platinum Mastermix (Thermo Scientific), 0.5 µL of each primer pair in a 50 pmol/µL primary concentration, and ultrapure nuclease-free water (Thermo Scientific) to achieve a final volume of 25 µL. The thermal cycle for the A, B, and C protocols was the same used for the identification of species and capsular type. Likewise, the visualisation of PCR products was obtained as already described.

### 2.3. Statistical Analysis

In order to conduct the statistical analysis, 2 × 2 contingency tables were constructed using Microsoft Excel^®^ version 16.0.17425.20176. The strains were classified in such tables to assess the association between the relevant findings and their detection/outcome, as described later. The 2 × 2 tables were constructed as shown in the following examples (Table 1, Table 2 and Table 3).

The derived figures were introduced in MedCalc software Odds ratio calculator© version 22.023 (https://www.medcalc.org/calc/odds_ratio.php; accessed on 6 April 2024). Odds ratio (OR), 95% confidence intervals (CI), and *p*-values were calculated to assess the association between: capsular types and virulence-associated genes; virulence-associated genes and lesions; and numbers of virulence-associated genes and lesions.

## 3. Results

### 3.1. Serogroup Distribution

A, D, and F were the serogroups identified among the tested strains (Table 4). Serogroup B was never detected. The serogroup most frequently identified was A, which was detected in 59.6% of tested strains. Serogroup F was detected in a rate of 22.8%, while serogroup D was found less frequently.

In the farms where it was possible to test a wider number of strains, the prevalent serogroup was A in rabbits from farms 1, 4, and 5, and F in rabbits from farm 7.

Concerning the serogroup distribution among the strains according to their origin, although with differences linked to the different farms, A was globally found in 62.2% of strains from rhinitis, while F and D were found in 21.62% and 16.21% of strains, respectively. Similarly, serogroups A, F, and D were identified in 35 (71.4%), 9 (18.36%), and 5 (10.2%) of strains from pneumonia, respectively. Among the strains isolated from the metritis, nine strains belonged to serogroup D and eight to serogroup A. Three strains only belonged to serogroup F. The strains from mastitis, abscesses, and septicaemia belonged to serogroup F, while those from otitis were identified as A.

### 3.2. Virulence-Associated Genes

The results concerning the finding of virulence-associated genes are reported in Table 5.

Gene *toxA* was not found in any of the tested strains. Among the genes encoding for the adhesion factors, those more frequently found were *pfhA* (73.78%), most frequently detected among serotypes A (76.27%) and F (100%), and *fim4* (92.23%) which was identified with a similar distribution among all the serogroups. Gene *tadD* was prevalently found in strains belonging to the serogroups A (59.32%) and D (57.89%), and *fimA* in strains belonging to types A (69.49%) and F (76%).

Concerning the genes encoding for extracellular enzymes, *sodC*, *nanB1*, and *nanH1* were identified in 103 (100%), 93 (90.29%), and 95 (92.23%) of the tested strains, respectively, while *sodA* was detected in 49 (47.57%) strains without relevant difference according to the serogroup belonging to.

Genes encoding for iron acquisition systems were less frequently found except for *fur* that was detected in 13 out 19 strains belonging to serogroup D (68.42%).

Among the genes associated with the membrane proteins *(OMPs)*, *oma87* was found in 99 out 103 (96.11%) tested strains with a similar high incidence among the different serogroups, while *plpB* was never detected.

Among the genes encoding for adhesion factors, *tadD*, *fim4*, and *fimA* were found with similar rates in the strains analysed independently to the lesion where they came from (Table 6). Instead, *pfhA* was prevalently found in strains from rhinitis (84.37%), pneumonia (77.27%), and other lesions (87.5%) in respect to those from metritis (42.1%).

No relevant differences according to the origin of the strains were found for genes encoding for extracellular enzymes, iron acquisition factors, and membrane proteins.

The distribution of virulence-associated genes contextually detected in the strains according to the lesion where they were identified is reported in Table 7.

Although most of them were provided of more than 7 virulence-associated genes, 15.6% of strains among those coming from rhinitis were provided up to 6 genes.

Strains detected in lungs affected by pneumonia were prevalently equipped with 7–8 genes (54.5%) and 9–11 genes (40.9%). Likewise, 68.4% and 21% of strains detected from metritis were provided of 7–8 genes and 9–11 genes, respectively. All strains isolated from abscess, otitis, mastitis, and septicaemia were found as provided of 7–8 genes (68.4%) or 9–11 genes (21%).

## 4. Discussion

According to previous studies [7,27,28], strains belonging to serogroup A were confirmed as the most frequently responsible for the disease in rabbits. In addition, the serogroup F, usually detected in turkeys and wild birds and found in rabbits also [29], was often identified. Although it was not considered a serogroup classically associated with pasteurellosis in rabbits for a long time, its incidence increased and was found exceeding the serogroup D in further studies [28,30]. Accordingly, serogroup F was more frequently detected than D among the tested strains. Even if with different distribution, strains belonging to different serogroups have been identified in the same holdings, confirming the wide spread of *P. multocida* and the evidence that different strains may be responsible for the disease occurring in a flock of rabbits in the same period and over the time. The spread of new serogroups may occur after the introduction, for managerial or commercial reasons, of new rabbits in the sheds and farms. Serogroup B, firstly reported in rabbits in India [9] and found also in a septicaemic rabbit in Italy [5], was never identified among the tested strains.

Although a strong relationship between capsular type and kind of lesion was not found, rhinitis and pneumonia were prevalently associated with the detection of type A, confirming that *P. multocida* strains belonging to serogroup A are frequently responsible for respiratory disorders in rabbits [31]. Strains identified from metritis prevalently belonged to serogroup A and D even if, in one farm, this lesion was also associated with strains belonging to A, D, and F.

Strains belonging to capsular type F were associated with abscesses, mastitis, and septicaemia, while serogroup A was found as responsible for otitis. Nevertheless, the limited number of tested strains from those lesions of rabbits coming from single farms does not provide comprehensive information and requires further investigation.

Although the capsule represents a pathogenicity factor of *P. multocida*, several virulence genes may play a key role determining the disease through different mechanisms of interaction with the host [31].

Among the genes encoding for adhesion factors, *pfhA* and *fim4* were the most detected according to previous studies [7,32]. Gene *pfhA*, encoding for a hemagglutinin and generally associated with disease in cattle, was globally detected in 73.8% of tested strains, according to previous studies involving strains from rabbit [7]. According to [33], *pfhA* was prevalently found in strains belonging to type A and less frequently in strains belonging to type D. Previously, *pfhA* was frequently detected in strains belong to serogroup F [28]. Interestingly, it was detected in all tested strains belonging to F, suggesting a strong relationship between *pfhA* and this capsular type, as it can be seen by the relevant OR > 1 and *p* < 0.05; as *phfA* was also detected in just less than a half of the tested strains belonging to D, this also suggests a strong relationship, as it can be seen by the relevant OR < 1 and *p* < 0.05, but rather suggesting that *pfhA* can be less frequently found in strains belonging to this serogroup. Gene *tadD* was rarely found in strains belonging to capsular type F, although that suggests a strong relationship between them, as it can be seen by the relevant OR < 1 and *p* < 0.05. Conversely, it was more frequently detected in type A and D strains, although of no statistical significance for both. Gene *tadD* was globally less frequently identified in contrast to previous studies involving *P. multocida* strains from rabbits [27,32]. *fim4* was detected in most of tested strains, while *fimA* was identified in approximately 65% of strains according to previous studies [27,32]. Gene *fimA*’s detection in tested strains belonging to D suggests a strong relationship between this gene and this serogroup, as it can be seen by the relevant OR < 1 and *p* < 0.05. According to the lesions where the strains came from, no relevant differences were found in the distribution of genes encoding for adhesion factors except for *tadD*, which was more frequently detected in strains responsible for metritis, and *pfhA* which was prevalently found in strains from the respiratory tract, although of no statistical significance for both these associations. However, *phfA*’s detection in tested strains isolated from metritis suggests a strong relationship between this gene and this lesion, as it can be seen by the relevant OR < 1 and *p* < 0.05 suggesting a possible reduced risk of this lesion. Among the genes encoding for enzyme production, *sodA* and *sodC* genes, which are linked to the production of superoxide dismutase enzymes, were previously detected in 100% of strains from rabbits [7]. Accordingly, *sodC* was identified in all tested strains but *sodA* was found less frequently similarly to more recent studies [27,32], respectively, without differences according to the lesions where they came from, but with a slight prevalence in strains belonging to type A and D.

According to the findings of [27,32], *nanH1* and *nanB1* were found in most strains, independently from the serogroup and kind of associated lesion, suggesting a relevant role in promoting colonisation and recognition of host receptors also. In fact, *nanH1* and *nanB1* encode to produce sialidases which are extracellular enzymes with glycolytic action that remove sialic acid from the glycolipids and glycoproteins of eukaryotic cells [34].

Concerning the genes encoding for iron acquisition mechanisms, the results were according to or in contrast with others, depending on the study [7,27,32]. *fur*, encoding for proteins involved in iron uptake regulation mechanisms and sporadically identified in strains from rabbits previously [27,32], occurred more frequently in our study, in strains belonging to serogroup D and involved with rhinitis, particularly, suggesting a strong relationship, as it can be seen by the relevant OR > 1 and *p* < 0.05, suggesting an increased risk of the gene presence for the occurrence of rhinitis due to strains belonging to serogroup D. In contrast, *exbB-tonB* was less frequently detected, regardless of capsular type and origin of the strain, than previously [7,27,32].

Gene *hgbB* was found according to [27,32] and, in our study, it was more frequently identified in strains responsible for metritis, suggesting a strong relationship as it can be seen by the relevant OR > 1 and *p* < 0.05 suggesting an increased risk of the occurrence of the lesion. Likewise, according to previous studies concerning *P. multocida* strains from rabbits, *tbpA* was rarely found. In fact, this gene, which encodes for the transferrin binding protein, is generally found in *P. multocida* strains from ruminants [7,35,36,37], and it is considered a marker of virulence for *P. multocida* strains responsible for disease, and associated with haemorrhagic septicaemia particularly, in cattle [35].

Genes encoding for iron uptake regulation mechanisms were not detected in strains identified from otitis, mastitis, septicaemia, and abscesses but as previously pointed out, the limited number of strains tested from these lesions does not allow for comprehensive information on this issue.

Among the genes associated with the OMPs, *oma87* encoding for membrane porins was found in 96.1% of tested strains, similarly to other studies [7], and a strong relationship between this gene and the occurrence of other lesions (i.e., mastitis, otitis etc.) was also found, as it can be seen by the relevant OR < 1 and *p* < 0.05 suggesting a possible reduced risk of this lesion; while *plpB* encoding for membrane lipoproteins was never detected. The genes associated with the OMPs are considered relevant in the pathogenesis of pasteurellosis because they interact with the host, promote the absorption of nutrients and act as a selective barrier against toxic molecules allowing the survival of the bacterium in different conditions [21].

Gene *toxA* was not found in tested strains belonging to A, according to other studies concerning *P. multocida* strains identified in rabbits [6,7] *Gene toxA* encodes for the dermonecrotic toxin which plays an important role in the pathogenesis of progressive atrophic rhinitis (PAR) of pigs [4]. In addition, the association of capsular type D strains with PAR or pneumonia was strongly correlated with the presence or absence of the *toxA* gene, respectively [38]. Although it was identified also in strains from cattle, small ruminants [7], and rabbits [39], a strong relationship between the strains equipped with *toxA* and clinical conditions was found in swine [7,38]. This could explain the lack of detection of *toxA* in strains isolated from purulent rhinitis and pneumonia investigated in our study. Most of the tested strains that came from rabbits which were sick or dead due to pasteurellosis, were equipped with seven to nine associated-virulence genes, suggesting that the association of more than one virulence genes may potentially increase the pathogenicity of a strain and influence the occurrence of clinical forms. Particularly, the presence of seven genes appears to be protective against the occurrence of rhinitis, as it can be seen by the relevant OR < 1 and *p* < 0.05, while it increases the occurrence of pneumonia, as it can be seen by the relevant OR > 1 and *p* < 0.05 suggesting an increased risk of the occurrence of the latter. The presence of eight genes increases the occurrence of metritis, instead, as it can be seen by the relevant OR > 1 and *p* < 0.05 suggesting an increased risk of the occurrence of this lesion. Some strains belonging to capsular type A, and responsible for respiratory disorders especially, were found equipped with 10 and 11 genes. Interestingly, among those, strains responsible only for rhinitis were also detected, suggesting that the pathogenic ability of the bacteria depends on the expression rather than the presence of a gene. The expression of the genes may be influenced also by different environmental conditions [40]. Accordingly, some strains with low potential pathogenicity because equipped with four to six genes were identified as responsible for rhinitis but also pneumonia and metritis, leading to consider the great relevance of the context in which the interaction between the germ and animal occurs. Therefore, although the magnitudes of gene expression may differ among isolates from different organs [41], the improvement of biosecurity measures and the correct management of rabbit flocks especially could play a fundamental role in influencing the pathogenic potential of *P. multocida* and the severity of the linked clinical forms. Additionally, the use of vaccines might be an additional method to control the infection.

## 5. Conclusions

Typing of *P. multocida* strains involved in case of pasteurellosis is important to assess their pathogenicity and better define the measures for disease control to apply in the affected rabbit flocks. Based on the results of this study, different associations of virulence-associated genes can potentially increase the pathogenic ability of *P. multocida* strains. Nevertheless, our findings also highlight that strains involved in less severe lesions can be found equipped with several virulence-associated genes. This finding suggests the relevance of the expression of a gene, rather than its presence, as responsible for the pathogenicity of the bacteria. Therefore, the improvement of the environmental conditions and sanitary management of rabbit flocks, and the increasing of biosecurity measures are relevant other than the investigation of the involved strains in case of *P. multocida* infection in rabbit farms.

## Figures and Tables

**Table 1:** Example of 2 × 2 table used to classify strains according to capsular types (detection) and virulence-associated genes (finding).

	Virulence-Associated Genes Concerned (Finding) e.g., *phfA*		
Capsular Types Concerned (Detection)	*phfA*	No-*phfA*	Total
Ser F	25	0	25
Non-ser F	51	27	78
Total	76	27	103

**Table 2:** Example of 2 × 2 table used to classify strains according to lesions (outcome) and virulence-associated genes (finding).

	Virulence-Associated Genes Concerned (Finding) e.g., *fur*		
Lesions Concerned (Outcome) e.g., Rhinitis	*fur*	No-*fur*	Total
Rhinitis	20	12	32
Non-rhinitis	24	47	71
Total	44	59	103

**Table 3:** Example of 2 × 2 table used to classify strains according to lesions (outcome) and numbers of virulence-associated genes (finding).

	Numbers of Virulence-Associated Genes Concerned (Finding) e.g., 8 Genes		
Lesions Concerned (Outcome) e.g., Pneumonia	8 Genes	Non-8 Genes	Total
Pneumonia	11	33	44
Non-pneumonia	23	36	59
Total	34	69	103

**Table 4:** Serogroup of *P. multocida* strains identified in lesions of rabbits from different farms.

		Farms									
		1	2	3	4	5	6	7	8	9	Total
Lesion	Serogroup	N° Belonging to/N° Tested (%)	N° Belonging to/N° Tested (%)	N° Belonging to/N° Tested (%)	N° Belonging to/N° Tested (%)	N° Belonging to/N° Tested (%)	N° Belonging to/N° Tested (%)	N° Belonging to/N° Tested (%)	N° Belonging to/N° Tested (%)	N° Belonging to/N° Tested (%)	N° Belonging to/N° Tested (%)
Rhinitis	A	13/22 (59.1)	1/2 (50)	-	5/9 (55.5)	-	4/4 (100)	-	-	-	23/37 (62.2)
	D	4/22 (18.2)	0/2 (0)	-	2/9 (22.2)	-	0/4 (0)	-	-	-	6/37 (16.2)
	F	5/22 (22.7)	1/2 (50)	-	2/9 (22.2)	-	0/4 (0)	-	-	-	8/37 (21.6)
Pneumonia	A	16/19 (84.2)	-	0/2 (0)	8/10 (80)	8/8 (100)	-	1/4 (25)	2/2 (100)	0 (0)	35/49 (71.4)
	D	3/19 (15.8)	-	2/2 (100)	0/10 (0)	0	-	0 (0)	0 (0)	0 (0)	5/49 (10.2)
	F	1/19 (5.3)	-	0/2 (0)	2/10 (20)	0	-	3/4 (75)	0 (0)	3/3 (100)	9/49 (18.4)
Otitis	A	-	-	-	2/2 (100)	-	-	-	-	-	2/2 (100)
	D	-	-	-	0/2 (0)	-	-	-	-	-	0/2 (0)
	F	-	-	-	0/2 (0)	-	-	-	-	-	0/2 (0)
Abscess	A	-	-	-	-	-	-	-	-	0/2 (0)	0/2 (0)
	D	-	-	-	-	-	-	-	-	0/2 (0)	0/2 (0)
	F	-	-	-	-	-	-	-	-	2/2 (100)	2/2 (100)
Mastitis	A	-	-	-	-	-	-	0/2 (0)	-	-	0/2 (0)
	D	-	-	-	-	-	-	0/2 (0)	-	-	0/2 (0)
	F	-	-	-	-	-	-	2/2 (100)	-	-	2/2 (100)
Septicaemia	A	-	-	-	-	-	-	0/2 (0)	-	-	0/2 (0)
	D	-	-	-	-	-	-	0/2 (0)	-	-	0/2 (0)
	F	-	-	-	-	-	-	2/2 (100)	-	-	2/2 (100)
Metritis	A	4/12 (33.3)	-	-	4/4 (100)	0/1 (0)	-	0/1 (0)	-	-	8/20 (40)
	D	8/12 (66.6)	-	-	0/4 (0)	1/1 (100)	-	0/1 (0)	-	-	9/20 (45)
	F	2/12 (16.6)	-	-	0/4 (0)	0/1 (0)	-	1/1 (100)	-	-	3/20 (15)
Total	A	33/56 (58.9)	1/2 (50)	0/2 (0)	19/25 (76)	8/9 (88.9)	4/4 (100)	1/9 (11.1)	2/2 (100)	0/5 (0)	68/114 (59.6)
	D	15/56 (26.8)	0/2 (0)	2/2 (100)	2/25 (8)	1/9 (11.1)	0/4 (0)	0/9 (0)	0/2 (0)	0/5 (0)	20/114 (17.5)
	F	8/56 (14.3)	1/2 (50.00)	0/2 (0)	4/25 (16)	0/9 (0)	0/4 (0)	8/9 (88.9)	0/2 (0)	5/5 (100)	26/114 (22.8)

**Table 5:** Virulence-associated genes detected in 103 *P. multocida* strains according to the serogroup belonging to.

Strain	Values	*toxA*	*pfhA*	*tadD*	*fim4*	*fimA*	*sodC*	*sodA*	*nanB1*	*nanH1*	*exbB-tonB*	*hgbB*	*fur*	*tbpA*	*oma87*	*plpB*
	N (%)	0/59 (0)	45/59 (76.27)	35/59 (59.32)	54/59 (91.52)	41/59 (69.49)	59/59 (100)	33/59 (55.93)	55/59 (93.22)	53/59 (89.83)	21/59 (35.59)	10/59 (16.94)	24/59 (40.67)	3/59 (5.08)	57/59 (96.61)	0/59 (0)
	OR	0.7479	1.3479	2.1065	0.7902	1.4342	1.3371	2.2212	2.1711	0.4206	2.4868	0.7937	0.8229	1.1250	1.3571	0.7479
Ser A	95% CI (min–max)	0.0146–38.4252	0.5576–3.2586	0.9519–4.6617	0.1785–3.4989	0.6305–3.2622	0.0260–68.6957	0.9971–4.9478	0.5736–8.2172	0.0807–2.1919	0.9780–6.3234	0.2921–2.1565	0.3740–1.8106	0.1799–7.0371	0.1836–10.0292	0.0146–38.4252
	*p*-value	0.8851	0.5074	0.0660	0.7565	0.3898	0.8851	0.0508	0.2536	0.3038	0.0557	0.6504	0.6280	0.8998	0.7647	0.8851
	N (%)	0/19 (0)	6/19 (31.57)	11/19 (57.89)	17/19 (89.47)	8/19 (42.10)	19/19 (100)	10/19 (52.63)	17/19 (89.47)	18/19 (94.73)	4/19 (21.05)	7/19 (36.84)	13/19 (68.42)	1/19 (5.26)	19/19 (100)	0/19 (0)
	OR	1.4274	0.2986	1.3750	0.6538	0.2909	0.2308	1.2821	0.8947	1.6364	0.6293	3.5000	3.7043	1.1111	2.1801	4.3333
Ser D	95% CI (min–max)	0.0560–36.3876	0.1033–0.8636	0.5027–3.7608	0.1214–3.5228	0.1042–0.8119	0.0044–11.9947	0.4729–3.4760	0.1742–4.5956	0.1892–14.1501	0.1899–2.0854	1.1481–10.6696	1.2782–10.7353	0.1171–10.5441	0.1126–42.2144	0.0834–225.2347
	*p*-value	0.8295	0.0257	0.5350	0.6210	0.0184	0.4669	0.6254	0.8940	0.6546	0.4487	0.0276	0.0159	0.9269	0.6062	0.4669
	N (%)	0/25 (0)	25/25 (100)	7/25 (28)	24/25 (96)	19/25 (76)	25/25 (100)	6/25 (24)	21/25 (84)	24/25 (96)	4/25 (16)	2/25 (8)	7/25 (28)	1/25 (4)	23/25 (92)	0/25 (0)
	OR	1.0131	27.2330	0.2705	2.3662	1.8741	0.3248	0.2570	0.4375	2.3662	0.4038	0.3120	0.4309	0.7708	0.3026	3.0784
Ser F	95% CI (min–max)	0.0440–25.6561	1.5960–464.6824	0.1013–0.7228	0.2768–20.2289	0.6716–5.2303	0.0063–16.7929	0.0926–0.7132	0.1128–1.6967	0.2768–20.2289	0.1253–1.3014	0.0668–1.4579	0.1618–1.1418	0.0821–7.2348	0.0404–2.2694	0.0595–159.1418
	*p*-value	0.9937	0.0224	0.0091	0.4315	0.2303	0.5764	0.0091	0.2319	0.4315	0.1288	0.1387	0.0921	0.8198	0.2449	0.5764
	Total N (%)	0/103 (0)	76/103 (73.78)	53/103 (51.45)	95/103 (92.23)	68/103 (66.01)	103/103 (100)	49/103 (47.57)	93/103 (90.29)	95/103 (92.23)	29/103 (28.15)	21/103 (20.38)	44/103 (42.71)	5/103 (4.85)	99/103 (96.11)	0/103 (0)

OR: Odds ratio; 95% CI: 95% confidence intervals (CI).

**Table 6:** Distribution of the virulence-associated genes in the tested strains according to the rabbit lesions.

Lesion	Values	*toxA*	*pfhA*	*tadD*	*fim4*	*fimA*	*sodC*	*sodA*	*nanB1*	*nanH1*	*exbB-tonB*	*hgbB*	*fur*	*tbpA*	*oma87*	*plpB*
	N (%)	0/32 (0)	27/32 (84.4)	14/32 (43.7)	30/32 (93.7)	19/32 (59.4)	32/32 (100)	18/32 (56.2)	28/32 (87.5)	30/32 (93.7)	9/32 (28.1)	8/32 (25)	20/32 (62.5)	2/32 (6.2)	31/32 (96.9)	0/32 (0)
	OR	2.2000	2.4245	0.6382	1.3846	0.6562	0.4545	1.6590	0.6462	1.3846	0.9978	1.4872	3.2639	1.5111	1.3676	2.2000
Rhinitis	95% CI (min–max)	0.0427–113.3326	0.8244–7.1302	0.2754–1.4789	0.2639–7.2659	0.2759–1.5607	0.0088–23.4158	0.7153–3.8478	0.1691–2.4689	0.2639–7.2659	0.3945–2.5241	0.5466–4.0466	1.3697–7.7777	0.2400–9.5155	0.1367–13.6784	0.0427–113.3326
	*p*-value	0.6950	0.1076	0.2949	0.7004	0.3406	0.6950	0.2383	0.5231	0.7004	0.9963	0.4371	0.0076	0.6601	0.7899	0.6950
	N (%)	0/44 (0)	34/44 (77.3)	23/44 (52.3)	40/44 (90.9)	32/44 (72.7)	44/44 (100)	20/44 (45.4)	41/44 (93.2)	40/44 (90.9)	14/44 (31.8)	5/44 (11.4)	16/44 (36.4)	2/44 (4.5)	43/44 (97.7)	0/44 (0)
	OR	1.3371	1.3762	1.0587	0.7273	1.7037	0.7479	0.8621	1.8397	0.7273	1.3689	0.3446	0.6327	0.8889	2.3036	1.3371
Pneumonia	95% CI (min–max)	0.0260–68.6957	0.5581–3.3935	0.4847–2.3127	0.1715–3.0836	0.7319–3.9659	0.0146–38.4252	0.3941–1.8857	0.4478–7.5591	0.1715–3.0836	0.5772–3.2467	0.1154–1.0286	0.2845–1.4066	0.1421–5.5602	0.2315–22.9267	0.0260–68.6957
	*p*-value	0.8851	0.4880	0.8862	0.6657	0.2165	0.8851	0.7102	0.3978	0.6657	0.4761	0.0562	0.2614	0.8998	0.4766	0.8851
	N (%)	0/19 (0)	8/19 (42.1)	12/19 (63.2)	18/19 (94.7)	12/19 (63.2)	19/19 (100)	8/19 (42.1)	17/19 (89.5)	18/19 (94.7)	6/19 (31.6)	8/19 (42.1)	8/19 (42.1)	1/19 (5.3)	19/19 (100)	0/19 (0)
	OR	4.3333	0.1711	1.7979	1.6364	0.8571	0.2308	0.7627	0.8947	1.6364	1.2241	3.9720	0.9697	1.1111	2.1801	4.3333
Metritis	95% CI (min–max)	0.0834–225.2347	0.0592–0.4945	0.6447–5.0142	0.1892–14.1501	0.3040–2.4170	0.0044–11.9947	0.2789–2.0863	0.1742–4.5956	0.1892–14.1501	0.4158–3.6035	1.3410–11.7649	0.3539–2.6572	0.1171–10.5441	0.1126–42.2144	0.0834–225.2347
	*p*-value	0.4669	0.0011	0.2623	0.6546	0.7707	0.4669	0.5978	0.8940	0.6546	0.7136	0.0128	0.9523	0.9269	0.6062	0.4669
	N (%)	0/8 (0)	7/8 (87.5)	4/8 (50)	7/8 (87.5)	5/8 (62.5)	8/8 (100)	3/8 (37.5)	7/8 (87.5)	7/8 (87.5)	0/8 (0)	0/8 (0)	0/8 (0)	0/8 (8)	6/8 (75)	0/8 (0)
	OR	11.2353	2.6377	0.9388	0.5568	0.8466	0.0890	0.6391	0.7326	0.5568	0.1326	0.2038	0.0681	0.9679	0.0645	11.2353
Other *	95% CI (min–max)	0.2095–602.6540	0.3093–22.4934	0.2217–3.9748	0.0597–5.1899	0.1902–3.7686	0.0017–4.7742	0.1445–2.8270	0.0808–6.6443	0.0597–5.1899	0.0074–2.3740	0.0113–3.6760	0.0038–1.2131	0.0492–19.0423	0.0077–0.5411	0.2095–602.6540
	*p*-value	0.2338	0.3751	0.9316	0.6072	0.8269	0.2338	0.5551	0.7821	0.6072	0.1699	0.2811	0.0675	0.9829	0.0115	0.2338

* Abscess, otitis, mastitis, septicaemia. OR: Odds ratio; 95% CI: 95% confidence intervals (CI).

**Table 7:** Distribution of the number of virulence-associated genes in the tested strains according to the rabbit lesions.

Lesion	Values	4 Genes	5 Genes	6 Genes	7 Genes	8 Genes	9 Genes	10 Genes	11 Genes
	N (%)	1 (3.1)	3 (9.4)	1 (3.1)	3 (9.4)	10 (31.2)	8 (25)	5 (15.6)	1 (3.1)
	OR	6.8095	3.5690	1.1129	0.2638	0.8902	1.1458	2.4444	1.1129
Rhinitis	95% CI (min–max)	0.2699–171.7976	0.5662–22.4958	0.0972–12.7380	0.0722–0.9644	0.3638–2.1780	0.4323–3.0372	0.6543–9.1323	0.0972–12.7380
	*p*-value	0.2441	0.1756	0.9315	0.0439	0.7988	0.7843	0.1838	0.9315
	N (%)	0	0	2 (4.54)	14 (31.8)	11 (25)	13 (29.5)	3 (6.8)	2 (4.5)
	OR	0.4382	0.1113	2.7619	2.5926	0.5217	1.8299	0.5436	2.7619
Pneumonia	95% CI (min–max)	0.0174–11.0149	0.0060–2.0686	0.2424–31.4704	1.0008–6.7165	0.2208–1.2328	0.7283–4.5975	0.1323–2.2333	0.2424–31.4704
	*p*-value	0.6160	0.1409	0.4131	0.0498	0.1381	0.1986	0.3978	0.4131
	N (%)	0	0	0	0	2 (100)	0	0	0
	OR	13.4000	3.5091	5.6286	0.6681	2.0606	0.6327	1.7429	5.6286
Otitis	95% CI (min–max)	0.4306–417.0271	0.1495–82.3523	0.2253–140.6111	0.0310–14.4091	0.1249–33.9835	0.0294–13.6315	0.0783–38.7984	0.2253–140.6111
	*p*-value	0.1390	0.4356	0.2926	0.7969	0.6132	0.7701	0.7257	0.2926
	N (%)	0	0	0	2 (100)	0	0	0	0
	OR	13.4000	3.5091	5.6286	18.7209	0.3913	0.6327	1.7429	5.6286
Abscess	95% CI (min–max)	0.4306–417.0271	0.1495–82.3523	0.2253–140.6111	0.8660–404.6878	0.0183–8.3790	0.0294–13.6315	0.0783–38.7984	0.2253–140.6111
	*p*-value	0.1390	0.4356	0.2926	0.0617	0.5484	0.7701	0.7257	0.2926
	N (%)	0	0	0	2 (100)	0	0	0	0
	OR	13.4000	3.5091	5.6286	18.7209	0.3913	0.6327	1.7429	5.6286
Mastitis	95% CI (min–max)	0.4306–417.0271	0.1495–82.3523	0.2253–140.6111	0.8660–404.6878	0.0183–8.3790	0.0294–13.6315	0.0783–38.7984	0.2253–140.6111
	*p*-value	0.1390	0.4356	0.2926	0.0617	0.5484	0.7701	0.7257	0.2926
	N (%)	0	0	0	0	1 (50)	1 (50)	0	0
	OR	13.4000	3.5091	5.6286	0.6681	2.0606	3.3913	1.7429	5.6286
Septicaemia	95% CI (min–max)	0.4306–417.0271	0.1495–82.3523	0.2253–140.6111	0.0310–14.4091	0.1249–33.9835	0.2041–56.3631	0.0783–38.7984	0.2253–140.6111
	*p*-value	0.1390	0.4356	0.2926	0.7969	0.6132	0.3944	0.7257	0.2926
	N (%)	0	2 (10.5)	0	2 (10.5)	11 (57.9)	2 (10.52)	2 (10.52)	0
	OR	1.4274	3.1765	0.5971	0.3529	3.6467	0.3316	1.1176	0.5971
Metritis	95% CI (min–max)	0.0560–36.3876	0.4925–20.4879	0.0296–12.0424	0.0752–1.6565	1.3028–10.2075	0.0708–1.5525	0.2176–5.7405	0.0296–12.0424
	*p*-value	0.8295	0.2243	0.7365	0.1868	0.0137	0.1610	0.8940	0.7365
	Total N (%)	1 (1)	5 (4.8)	3 (2.9)	23 (22.3)	34 (33)	24 (23.3)	10 (9.7)	3 (2.9)

OR: Odds ratio. 95% CI: 95% confidence intervals (CI).

## Data Availability

The original contributions presented in the study are included in the article.
